# Early Intervention in Cognitive Aging with Strawberry Supplementation

**DOI:** 10.3390/nu15204431

**Published:** 2023-10-19

**Authors:** Robert Krikorian, Marcelle D. Shidler, Suzanne S. Summer

**Affiliations:** 1 Department of Psychiatry & Behavioral Neuroscience, University of Cincinnati Academic Health Center, Cincinnati, OH 45267, USA; shidlemd@ucmail.uc.edu; 2 Bionutrition Core, Schubert Research Clinic, Cincinnati Children’s Hospital Medical Center, Cincinnati, OH 45229, USA; suzanne.summer@cchmc.org

**Keywords:** strawberries, anthocyanins, insulin resistance, aging, cognition, dementia risk reduction

## Abstract

Late-life dementia is a growing public health concern lacking effective treatment. Neurodegenerative disorders such as Alzheimer’s disease (AD) develop over a preclinical period of many years beginning in midlife. The prevalence of insulin resistance, a prominent risk factor for late-life dementia, also accelerates in middle-age. Consumption of berry fruits, including strawberries, has been shown to influence metabolism as well as cognitive performance suggesting potential to mitigate risk for dementia. In this controlled trial, we enrolled overweight middle-aged men and women with insulin resistance and subjective cognitive decline and performed a 12-week intervention with daily administration of whole-fruit strawberry powder. Diet records showed that participants in both groups maintained the prescribed abstinence from berry product consumption outside the study. We observed diminished memory interference (*p* = 0.02; Cohen’s *f* = 0.45) and a reduction of depressive symptoms (*p* = 0.04; Cohen’s *f* = 0.39) for the strawberry-treated participants; benefits consistent with improved executive ability. However, there was no effect of the intervention on metabolic measures, possibly a consequence of the sample size, length of the intervention, or comparatively low anthocyanin dose. Anti-inflammatory actions of anthocyanins were considered as a primary mechanistic factor. The findings support the notion that strawberry supplementation has a role in dementia risk reduction when introduced in midlife. However, further investigation with longer intervention periods, larger samples, and differing dosing regimens will be required to assess the benefits of strawberry intake with respect to cognition and metabolic function in the context of aging.

## 1. Introduction

Alzheimer’s disease (AD) is the most common form of dementia, accounting for up to 80% of cases, and current projections indicate that AD will reach epidemic proportions during the next several years [1]. Paralleling the increase in dementia prevalence is an equally disturbing rise in metabolic disturbance reflected in insulin resistance, hyperinsulinemia, obesity, and related health conditions [2] associated with late-life dementia [3,4]. There is no remedy for dementia, and it is not clear when or if effective therapy will be available. Accordingly, prevention and mitigation of risk have been increasingly emphasized.

Alzheimer’s neuropathology is thought to develop and progress during a period of many years prior to clinically evident impairment [5]. This extended preclinical period represents an opportunity for intervention to lower risk for progressive decline. Demographically, the preclinical phase of accelerating AD pathology corresponds with the midlife epoch during which metabolic disturbance also becomes prominent [6,7]. The percentage of US adults classified as metabolically healthy by common standards is exceedingly small [8], and nearly 50% of middle-aged adults in the US have insulin resistance [6]. There is increasing evidence that insulin resistance and associated hyperinsulinemia and obesity are important drivers of early neurodegenerative changes [9,10]. Risk for AD, Parkinson’s disease, and other dementing disorders is elevated in the context of metabolic disturbance [11,12,13], which accelerates beta-amyloid deposition and tau hyperphosphorylation, characteristic neuropathological features of AD [14].

Metabolic disturbance and obesity create an environment that supports chronic inflammation, affecting many organs including the brain [2]. Neuroinflammation is recognized as one of the more important factors contributing to brain dysfunction and progressive neuropathology [15,16]. Berry fruit supplementation has the potential to confer multiple health benefits such as moderation of oxidative stress and inflammation [17], correction of metabolic dysfunction [18,19,20], and augmentation of neuronal signalizing [21]. Anthocyanins and their metabolites, which are present in strawberries and blueberries among other fruits and vegetables, have been shown to have anti-inflammatory actions [16]. In addition, they have been identified in the brain as well as in peripheral organs, indicating ability to cross the blood–brain barrier [22]. Further, modification of the gut microbiota following anthocyanin consumption can alter peripheral–central communications to mitigate central inflammation [23].

In a recent cross-over trial involving obese, insulin-resistant adults, high-dose daily strawberry supplementation for four weeks lowered fasting insulin and the homeostasis model assessment of insulin resistance (HOMA-IR) [24]. These effects were attributed, at least in part, to anthocyanin-related lowering of circulating levels of branch chain amino acids, which has been associated with diminished risk for type 2 diabetes [25,26]. In a controlled, acute response study with overweight men and women, whole fruit strawberry powder containing 39 mg anthocyanins consumed with a high carbohydrate, high fat meal reduced plasma insulin and postprandial inflammatory markers including C-reactive protein and IL-6. These effects were associated with increased levels of anthocyanins and metabolites in postprandial plasma [27]. In addition, in controlled studies with overweight and hypercholesteremic adults involving chronic and acute ingestion of whole fruit strawberry powder, beneficial changes in vascular, lipid, and metabolic markers were observed [28,29].

In addition to benefits with respect to vascular, lipid, and metabolic markers, there are preliminary indications of improved neurocognitive function with strawberry supplementation. Preclinical data suggest that strawberry supplementation can improve cognitive performance and markers of neuronal function [30,31,32], and prospective epidemiological evidence indicates that habitual consumption of strawberries (and blueberries) is associated with diminished rate of age-related cognitive decline [33] and with lower risk for Alzheimer’s disease, specifically [34]. Further, a recent controlled trial with older men and women showed that 90 days’ supplementation with two servings of whole fruit, freeze-dried strawberry powder improved cognitive performance in terms of decreased response latency during a spatial navigation task and improved recognition memory on a test of word list learning [35]. In another cross-over trial, eight weeks’ supplementation with two servings of strawberry powder was associated with enhanced speed of cognitive processing as well as lower blood pressure, reduced waist circumference, and increased antioxidant capacity [36].

Given the evidence regarding benefits for metabolic function, general health, and cognitive performance, we sought to investigate the effects of strawberry supplementation on neurocognitive function when instituted in the midlife preclinical period as an initial investigation of early intervention for dementia risk mitigation. We assessed the effects of strawberry supplementation in middle-aged individuals with increased risk for future cognitive decline. The primary outcomes included measures of neurocognitive domains vulnerable to dementia including executive ability, lexical access, memory, and mood. Secondarily, we assessed whether strawberry supplementation was associated with improvement in metabolic and anthropometric parameters. 

## 2. Materials & Methods

Study design: The study was conducted in accordance with the Declaration of Helsinki and approved by the University of Cincinnati Medical Institutional Review Board (protocol 2015-1256; 8 March 2023). It was registered with Clinical Trials Identifier NCT02751866. 

This was a randomized, double-blind, placebo-controlled trial. Assessments were performed prior to and after 12 weeks’ supplementation. Primary outcome measures included neurocognitive and mood measures. We also assessed metabolic and anthropometric parameters. Three-day diet records were obtained pre- and post-intervention to monitor flavonoid consumption external to the study. 

**Figure 1:**
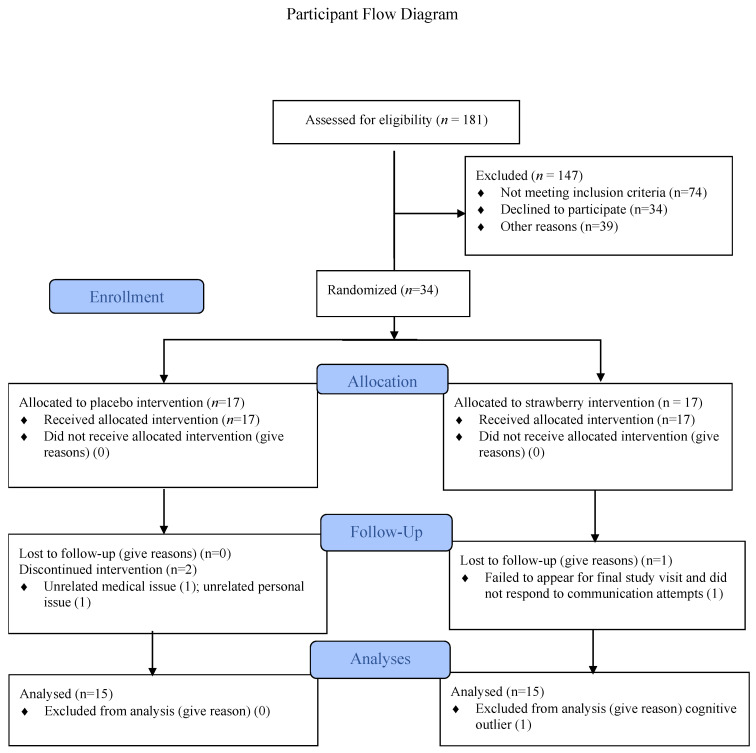
Participant flow diagram showing data concerning participant screening, enrollment, group allocation, completion, and number of participants included in the analyses.

Participants: Overweight, middle-aged men and women with complaints of mild cognitive decline were recruited by means of advertising placed on the University of Cincinnati Academic Health Center website, flyers displayed in medical clinics, and email messages to employees of the affiliated Cincinnati Children’s Hospital Medical Center describing the research opportunity. Figure 1 shows data concerning participant recruitment, screening, enrollment, and non-completion. A total of 34 were enrolled in the study; 17 in each group.

Inclusion criteria: (1) Men and women 50 to 65 years old; (2) BMI = 25 or greater; (3) awareness of mild cognitive decline; (4) ability to comprehend and comply with the research protocol; (5) provision of written informed consent.

Exclusion criteria: (1) Diagnosis of neurological disorder, dementia, or memory disorder such as mild cognitive impairment, probable Alzheimer’s disease, Parkinson’s disease, frontotemporal dementia; (2) current or past psychiatric condition or substance use causing a persisting change in level of functioning; (3) diagnosis of diabetes or other metabolic disorder or kidney or liver disease; (4) regular use of medication or dietary supplement that might affect outcome measures such as benzodiazepine, psychostimulant, and berry fruit extract.

Telephone screening: We performed an initial telephone contact in which a description of the requirements for study participation was provided. We also obtained oral informed consent and administered the Academic and Medical History Questionnaire [37] to acquire demographic, educational, and medical information and to estimate body mass index (BMI) using height and body weight information provided by the prospective participants. In addition, we administered the modified Memory Impairment Screen (mMIS) [38] to identify those with greater than mild memory decline for exclusion from the study. For those who qualified for study participation, BMI was calculated with anthropometric measurements at the subsequent enrollment visit. We instructed qualifying participants to refrain from consumption of all berry fruits and products for at least two weeks prior to the enrollment study visit and to complete a three-day diet record during the week prior to enrollment. 

Enrollment visit: All participants reviewed and signed the written informed consent document at this study visit. This was followed by review of the diet records that had been completed during the prior week. We collected fasting blood samples, measured anthropometric parameters, administered neuropsychological measures and a mood inventory (see descriptions below), and provided a supply of strawberry or placebo powder. 

Interim visit: The participants returned during week 6 of the intervention when we collected used and unused supplement packets and distributed the final 6-week supply of packets along with the diet record forms to be completed during the week before the final study visit.

Final visit: During the final visit, we re-administered the measures obtained at the enrollment study visit and again obtained fasting blood samples.

### 2.1. Strawberry Powder, Placebo Powder, and Supplement Regimen

We utilized strawberry powder and placebo powder supplied for this research by the California Strawberry Commission, Watsonville, California, USA. The strawberry powder was prepared from whole fruit that had been desiccated, freeze-dried, and milled. The placebo was designed to have the same appearance, taste, and carbohydrate load as the strawberry powder and contained fiber but no polyphenolic content. Daily servings of strawberry and placebo powder were sealed in packets for the convenience of the participants and to control daily dosage. Each packet of strawberry powder contained 13 g, providing 36.8 mg anthocyanins derived from 130 g whole fruit and equivalent to about 1 c whole fresh strawberries, which is designated as a standard serving by the California Strawberry Commission.

We instructed participants to mix the contents of one packet with water and consume it with the first meal of the day, although taking the powder with other foods or beverages was not prohibited. We also asked participants to discontinue consumption of all berry fruits, juices, and extracts for the duration of the study and provided a list of forbidden berry products. This was done to mitigate the potential confound related to a group difference in consumption of berry products in the background diet. We did not attempt to control polyphenol consumption from non-berry fruits and vegetables as we judged this to be unreasonably burdensome and not representative of general consumption habits. However, we measured anthocyanin consumption external to the study with diet diary records completed by the participants. This allowed assessment of adherence to the prescription against berry intake outside the study and the means to assess potential differential intake between the groups.

Randomization and research supplement: Participants were assigned to one of two intervention groups using the method of Taves [39] to achieve block group assignment. The investigators and participants were blind to group membership. The powder packets were labeled with a numeric code and stored in a cold room until dispensed to the participants with instructions to refrigerate at home until consumed. We collected unused and used packets at subsequent study visits as a check on compliance with the intervention protocol.

### 2.2. Outcome Measures and Assessment Procedures

The primary outcomes included cognitive and mood measures. The neurocognitive protocol included tasks assessing executive abilities including inhibitory control and task switching, lexical access, long-term memory function, and mood; domains implicated in both non-pathological aging and late-life dementia. Equivalent, alternate forms of the long-term memory tasks were employed to minimize practice effects associated with repeat exposure to specific test items. The cognitive protocol was administered at the enrollment and final (week 12) study visits.

### 2.3. Executive Abilities

The Porteus Maze Test (PMT) is a paper and pencil task that requires participants to draw continuous pencil lines to solve a series of two-dimensional mazes of increasing complexity [40]. This problem-solving task requires planning and cognitive and motor control and has predictive value with respect to functional capability and ability to adapt and cope with life challenges [40,41]. We used a series of mazes drawn from the three forms of the PMT, and alternate versions were administered at the baseline and final study visits. 

Trail-Making Test, part B was used to evaluate working memory and set switching aspects of executive ability. The Trail-Making Test, part B, involves alternate sequencing of a two-dimensional array of digits and letters by drawing pencil lines as quickly as possible [42,43]. 

### 2.4. Lexical Access

Controlled Oral Word Production: Lexical access refers to the ability to retrieve information from the mental word store (lexicon) and depends on previously acquired word knowledge and phonemic and semantic information processing [44] as well as executive control processes such as updating, shifting mental set, and inhibition [45]. The task is administered in two trials with phonemic and categorical constraints, respectively, and yields a score for each trial; the number of words produced that begin with specified letters of the alphabet and the number of words produced that are representative of a semantic category [46].

### 2.5. Learning and Long-Term Memory

The California Verbal Learning Test, Second Edition (CVLT; [47]) is a word list learning and retention task that assesses verbal learning and long-term memory function. It yields measures of cumulative learning across exposure trials and measures of recall and recognition memory. In addition, it provides data on interference during learning and recall evident as intrusion responses on recall and recognition testing that reflect interference of non-target words. 

The Spatial Paired Associate Learning Test (SPAL) assesses ability to learn associations of visual-spatial stimuli that are presented as pairs during learning trials [48]. The test items are designed to be non-representative and to resist verbal encoding. It is a challenging nonverbal memory task, in part because it requires reproduction rather than merely recognition of pairs of abstract visual configurations. 

### 2.6. Mood Symptoms

The Beck Depression Inventory-II was administered to obtain a quantitative assessment of the intensity of depressive symptoms such as hopelessness, irritability, and guilt [49,50]. It was administered at the enrollment and final study visits to measure change in mood symptoms during the intervention. We excluded individuals with high scores on the BDI, suggesting the presence of clinically significant mood disturbance that might influence cognitive performance and/or ability to engage in the intervention [51].

### 2.7. Metabolic Parameters

Fasting blood samples were assayed at the Mouse Metabolic Phenotyping Laboratory at the University of Cincinnati (UC) Metabolic Diseases Research Center. Assays were performed for glucose, insulin, and glycated hemoglobin, a marker of average plasma glucose over a 90-day period. Glucose and insulin values were used to calculate the homeostasis model assessment of insulin resistance (HOMA2-IR) [52]. We also obtained triglycerides (TG) and high-density lipoprotein cholesterol (HDL-C) values and calculated the TG over HDL-C ratio, which is associated with insulin resistance, coronary artery disease, and with features of the metabolic syndrome [53,54,55].

### 2.8. Anthropometric Measures

We measured body weight, waist circumference, and height at the enrollment visit and body weight and waist circumference at the final visit. 

### 2.9. Diet Diaries

We obtained records of all food and beverage consumption for three-day periods during the week before enrollment and the week before the final visit to monitor the background diet during the intervention. These diet diaries were completed by the participants who were given oral and written instruction regarding accurate completion of the records. The Nutrition Data Systems for Research software, version 2019 (Nutrition Coordinating Center, University of Minnesota, Minneapolis, MN, USA) was used to analyze total energy, macronutrient proportions, and anthocyanin intake at the Bionutrition Core of the Cincinnati Children’s Hospital Medical Center’s Schubert Research Clinic. 

### 2.10. Statistical Analyses and Power Calculations

Data from our prior research in this population [56] indicated that berry intervention studies produced medium to large effects on neurocognitive and metabolic factors. Accordingly, we planned the study assuming medium effect sizes. Given this assumption and resource limitations for this research, we calculated that a sample size of 30 participants with 15 per group would be sufficient to detect effects with power = 0.75 and *α* = 0.05 [57]. 

We investigated effects of the intervention on measures of executive ability, lexical access, verbal and nonverbal memory function, and interference in memory. Using baseline test scores, we examined inter-correlations within and between measures for each domain to corroborate inclusion of specific measures in representative composite scores. Test scores were standardized, and means were calculated to create the domain scores [58]. The executive domain composite included the Porteus Maze Test and Trail-Making Test, part B. The lexical access composite incorporated the phonological and semantic production scores from the Controlled Oral Word Association task. The verbal memory domain included the CVLT cumulative learning score and the CVLT long-delay recall score. The visual-spatial memory domain included the SPAL learning and recognition memory scores. The CVLT total intrusion score represented memory interference. The BDI total score represented the mood domain. 

The hypotheses of beneficial neurocognitive effects were tested with separate ANCOVAs comparing cognitive and mood domain final visit scores between groups with covariate control for the corresponding enrollment measures [59]. We also calculated Cohen’s *f* statistic to generate effect size estimates for statistical effects at *p* ≤ 0.05. Cohen’s *f* represents the effect size estimate implemented in ANOVA analyses that derive the *F*-test and is an extension of the effect size statistic *d* [57]. Cohen’s *f* values are described as ranging from small (0.10) to medium (0.25) and large (0.40) [57]. In this study, effect sizes were large, and power to detect differences was greater than 0.75.

## 3. Results

The sample contained a total of 30 participants including 5 males and 25 females. There were 5 males and 10 females in the placebo group, and all participants in the strawberry group were female. There was no pre-intervention difference between males and females with respect to demographic factors or performance on the cognitive screening measure (mMIS). Table 1 contains demographic, cognitive, anthropometric, and metabolic information for the participant sample at the time of enrollment. Group comparisons of these pre-intervention data showed that the groups were comparable with respect to age, education, and general memory performance data. While the mean fasting glucose value for the strawberry-treated group was lower that of the placebo group, *p* = 0.02, there was no pre-intervention group difference with respect to the other anthropometric and metabolic measures. Mean group values for body weight, BMI, and waist circumference reflected early metabolic disturbance [60] in accordance with the inclusion criteria. This was corroborated further by the mean HOMA2-IR [61] and glycated hemoglobin values [62], which were below the threshold for diabetes but in the high normal and low prediabetes range, respectively. Similarly, both groups exhibited mean TG/HDL-C ratios that reflected higher than optimal values but below the threshold for type 2 diabetes [63].

**Figure 2:**
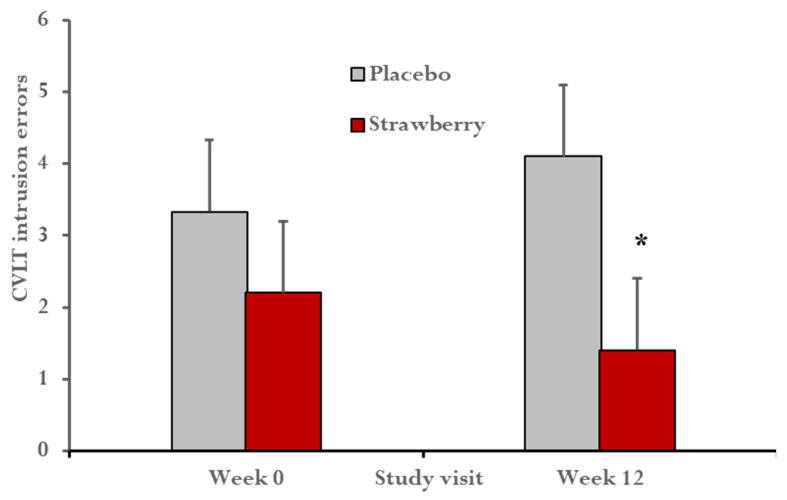
After 12 weeks, the strawberry-treated group exhibited fewer intrusion errors on the CVLT, a measure of interference during learning and memory. Lower scores represent better performance. * *F*(1,27) = 5.69, *p* = 0.02, Cohen’s *f* = 0.45. Error bars = SEM.

**Figure 3:**
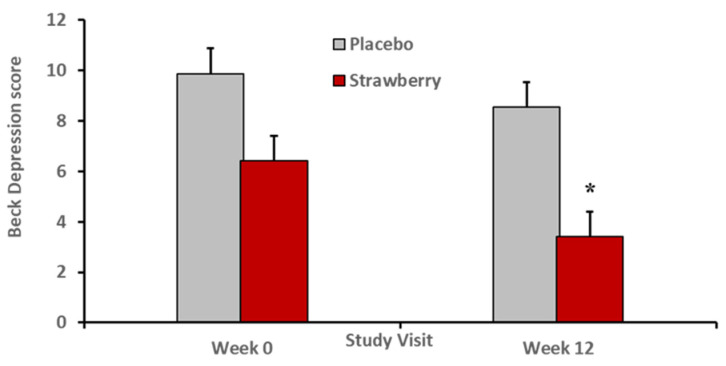
After 12 weeks, the strawberry-treated group reported a lower level of depressive symptoms on the Beck Depression Inventory. * *F*(1, 27) = 4.28, *p* = 0.04, Cohen’s *f* = 0.39. Error bars = SEM.

ANCOVAs examining group differences for cognitive domains indicated that strawberry supplementation did not improve performances with respect to the executive domain, *p* = 0.71, lexical access domain, *p* = 0.14, verbal memory, *p* = 0.69, or visual-spatial memory, *p* = 0.76. However, the strawberry-treated group exhibited a relative reduction of interference in verbal learning and memory *F*(1,27) = 5.69, *p* = 0.02, Cohen’s *f* = 0.45 (Figure 2). In addition, the strawberry group reported lower mood disturbance relative to the placebo group, *F*(1, 27) = 4.28, *p* = 0.04, Cohen’s *f* = 0.39 (Figure 3). 

With respect to metabolic measures, there was no intervention effect for fasting glucose, *p* = 0.61, fasting insulin, *p* = 0.29, HOMA2- IR, *p* = 0.45, glycated hemoglobin, *p* = 0.67, or the TG/HDL ratio, *p* = 0.85, *p* = 0.43. Similarly, anthropometric measures including body weight, *p* = 0.17 and waist circumference, *p* = 0.34 were not affected, although there was a weak trend favoring the strawberry-treated group with respect to lower BMI, *p* = 0.10. 

**Figure 4:**
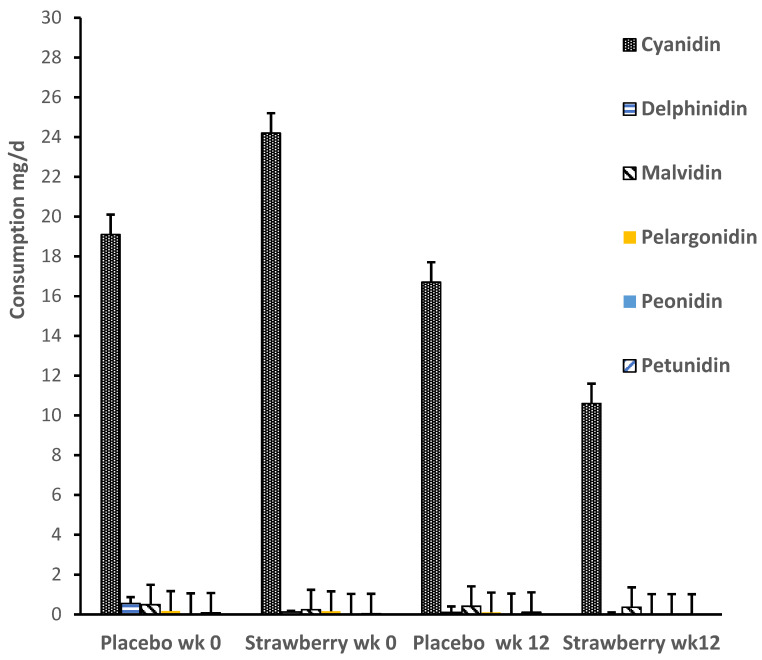
Mean daily consumption by group for each of the major anthocyanins prior to (wk 0) and during the final week of the study (wk 12). A repeated measures ANOVA indicated that there was no between-group difference in anthocyanin consumption external to the study, *F*(1,27) = 1.26, *p* = 0.26, and no change in consumption during the period of intervention *F*(1,27) = 0.61, *p* = 0.43. Error bars = SEM. wk = week.

Diet diary data are shown in Figure 4. A repeated measures ANOVA indicated that there was no between-group difference in anthocyanin consumption in the background diet during the intervention, *p* = 0.26. In addition, there was no group by time interaction, *p* = 0.43, indicating no change in background anthocyanin consumption during the period of the intervention. As shown in Figure 4, cyanidin consumption external to the study was much greater than any other anthocyanin, which is consistent with our prior berry supplement studies in this population [56] and almost certainly attributable to the extensive presence of cyanidin in many non-berry fruits and vegetables [64].

## 4. Conclusions

In this controlled pilot trial, overweight middle-aged participants treated with whole-fruit strawberry powder for 12 weeks exhibited fewer intrusion errors on a word list learning task, reflecting reduction of interference of extraneous information during learning and recall. Such interference typically involves an inability to inhibit intrusion of competing exemplars within a semantic category [65]. The finding of improvement in this regard for the strawberry-treated group might be understood as reflecting more effective executive control processes supporting suppression of non-target terms [66]. Memory interference is not uncommon in the context of aging and, especially in late life dementia [66,67], has been associated with greater regional neurodegeneration [67].

In addition, we observed that members of the strawberry-treated group endorsed lower levels of depressive symptoms. This relative mood enhancement experienced by the strawberry-treated group implies improved emotional coping capability and lower levels of stress [68]. Such coping enhancement also can be understood as implying improved executive ability; that is, better ability to manage everyday activities and social relationships and improved response control and greater flexibility [69]. Notably, the role of executive capability in coping, stress control, and mood disturbance has been documented in the context of clinical depression as well [68,69].

Demographically, executive capability begins to decline in middle-age [70]. Further, executive decline is exacerbated in the context of obesity and insulin resistance [71,72]. Therefore, it is likely that for many or all participants in our sample of insulin resistance, middle-aged participants executive ability was at least mildly impaired as a consequence of age and metabolic status. Presumably, such executive deficits also contributed to the subjective perception of mild cognitive decline, =a criterion for inclusion in the study. This would imply that the strawberry treatment corrected, to a measurable extent, existing executive deficiency,. It is of interest to note that failure to recover from proactive semantic interference on word list learning tests is an early marker of AD pathology in older adults [67].

Reduction of memory interference also was observed in a supplementation trial with blueberries in the same population; insulin-resistant, middle-aged individuals [56], corroborating the notion that anthocyanin supplementation tends to improve deficits associated with aging, insult, ill health, and fatigue, but generally has little effect on preserved cognitive capabilities. That is, benefits tend to be limited to deficient functions while unimpaired capabilities are not enhanced.

Chronic metabolic disease, in particular peripheral hyperinsulinemia [9] and obesity [73], strongly influence brain function and risk for neurodegeneration disease. Adipocyte-generated inflammation in overweight and obese individuals is a primary driver of cerebral atrophy [74]. Recent data from a very large cross-sectional study demonstrated strong associations of regional and whole brain atrophy with subcutaneous, and especially visceral, fat deposition in middle-aged and older men and women [75], supporting the notion that excess abdominal fat is pro-inflammatory and has implications for brain integrity and neurodegeneration [16]. In addition, there is evidence that cellular energy production in the brain is inversely correlated with BMI, even in young healthy adults [76]. These observations indirectly implicate anthocyanin-related anti-inflammatory effects among the mechanistic factors involved in reducing brain dysfunction [77], with both peripheral and central actions of anthocyanins and metabolites mitigating neuroinflammation [15,16,22,23].

It is notable that our inclusion and exclusion criteria were successful in recruiting a sample of individuals with insulin resistance. However, counter to our expectation we did not observe improvement for the strawberry-treated group in metabolic measures including fasting insulin, HOMA-IR, TG/HDL-C ratio, and glycated hemoglobin. This fact, and the absence of change in anthropometric measures, suggest the possibility that the cognitive and mood effects were not attributable to enhancement of metabolic function as we had expected. It may be that anti-inflammatory actions of anthocyanins were effective in correcting aspects of neurocognitive deficit in the absence of improvement of metabolic health. 

Methodological factors that might account for the absence of change in metabolic markers would include the length of the intervention, the relatively small sample size, and the anthocyanin dose provided to the strawberry group. Anthocyanin representation in strawberries is appreciably lower than that found in blueberries [78]. Specifically, the anthocyanin concentration of the standard 13 g strawberry powder serving used in this study was 36.8 mg, while the anthocyanin concentration in 10 g blueberry powder (about 0.5 c whole fruit equivalent) provides 140 mg anthocyanins. While some of the single dose, acute response studies have employed dosages as low as 10 g strawberry powder [27], the majority of the chronic feeding studies have used higher daily dosages such as 26 g and 32.5 g, providing 73.6 mg and 92 mg anthocyanins, respectively and have demonstrated benefits with respect to metabolic, lipid, and other markers [28,29], supporting the notion that higher anthocyanin intake in chronic feeding studies may be necessary to achieve change in metabolic parameters. Metabolic benefits, including reduced fasting insulin and lower HOMA-IR, were demonstrated in a trial comparing higher dose (32 g daily strawberry supplementation) against lower dose (13 g daily supplementation) and control powders. Only the higher dose strawberry powder regimen providing 92 mg anthocyanins was effective [25]. In that study, the lower, ineffective dosage providing 36.8 mg anthocyanin daily was the same dosage administered in this trial. Also, as noted, neurocognitive benefit has been observed with chronic daily feeding at 25 g powder containing 73.6 mg anthocyanin [35].

An early, seminal animal study showed that supplementation with fruit extracts with high antioxidant activity including strawberries and blueberries (as well as spinach) fed to aging rats was effective in reversing functional decrements of motor function, cognitive performance, and several measures of neuronal function to levels observed in young animals [30]. In that study, blueberry extract supplementation was most effective with respect to certain measures of neuronal function, and the only extract associated with restoration of motor capability [30]. Early animal research also demonstrated the presence of anthocyanins in specific brain regions mediating cognitive and motor function following supplementation with blueberry [79,80]. Further, supplementation with pure anthocyanins as well as whole blueberry extracts was protective against brain insults such as irradiation [81,82] and poor nutrition [83]. In addition, a recent human trial demonstrated strong association of cognitive benefits with urinary anthocyanin metabolites in older adults receiving blueberry supplementation [84].

In summary, this controlled trial showed that daily supplementation with 13 g whole fruit strawberry powder reduced interference in memory and depressive symptoms in overweight middle-aged individuals. These findings were understood as manifestations of improved executive control. Anti-inflammatory action of anthocyanin-containing strawberry powder was suggested as a primary mechanistic factor. The unexpected absence of benefit with regard to metabolic function might reflect the lower dose of anthocyanins used in this study as compared with other trials investigating metabolic and cognitive function. Other limitations included the sample size and length of the intervention. These considerations highlight the need for further investigation of health and neurocognitive benefits associated with strawberry supplementation employing different dosages, larger samples, and intervention periods of varying lengths.

## Figures and Tables

**Table 1:** Participant sample characteristics at enrollment.

	Placebo (*n* = 15)	Strawberry (*n* = 15)	*t*; *p*
Age, y	57.0 (3.9)	55.9 (4.7)	0.70; 0.48
Education, y	16.2 (1.7)	15.4 (1.9)	1.08; 0.28
mMIS	12.2 (1.3)	12.4 (2.0)	0.27; 0.78
Body weight, kg	101.1 (15.4)	99.2 (24.3)	0.25; 0.79
BMI	37.2 (6.2)	35.1 (8.6)	0.78; 0.44
Waist circumference, cm	112.6 (13.4)	115.5 (22.0)	0.43; 0.67
Fasting glucose, mg/dL	113.2 (14.3)	101.8 (12.2)	2.32; 0.02
Fasting insulin, µU/mL	12.8 (9.6)	11.4 (5.2)	0.50; 0.62
HOMA2-IR	3.6 (2.8)	2.9 (1.5)	0.82; 0.41
HbA1c, %	5.8 (0.52)	5.6 (0.27)	1.27; 0.26
TG/HDL-C ratio	1.91 (0.99)	1.89 (1.02)	1.04; 0.96

Note. Mean (SD) by group. mMIS = modified Memory Impairment Screen [38]. BMI = body mass index. HOMA2-IR = homeostasis model assessment of insulin resistance. HbA1c = glycated hemoglobin. TG = triglycerides. HDL-C = high density lipoprotein cholesterol.

## References

[1] Rajan K.B., Weuve J., Barnes L.L., McAninch E.A., Wilson R.S., Evans D.A. (2021). Population estimate of people with clinical Alzheimer’s disease and mild cognitive impairment in the United States (2020–2060). Alzheimer’s Dement..

[2] Saltiel A.R., Olefsky J.M. (2017). Inflammatory mechanisms linking obesity and metabolic disease. J. Clin. Investig..

[3] Livingston G., Sommerlad A., Orgeta V., Costafreda S.G., Huntley J., Ames D., Ballard C., Banerjee S., Burns A., Cohen-Mansfield J. (2017). Dementia prevention, intervention, and care. Lancet.

[4] Livingston G., Huntley J., Sommerlad A., Ames D., Ballard C., Banerjee S., Brayne C., Burns A., Cohen-Mansfield J., Cooper C. (2020). Dementia prevention, intervention, and care: Report of the Lancet Commission. Lancet.

[5] Sperling R.A., Aisen P.S., Beckett L.A., Bennett D.A., Craft S., Fagan A.M., Iwatsubo T., Jack C.R., Kaye J., Montine T.J. (2011). Toward defining the preclinical stages of Alzheimer’s disease: Recommendations from the national institute on aging-Alzheimer’s association workgroups on diagnostic guidelines for Alzheimer’s disease. Alzheimer’s Dement. J. Alzheimer’s Assoc..

[6] Hirode G., Wong R.J. (2020). Trends in the prevalence of metabolic syndrome in the United States, 2011–2016. JAMA Lett..

[7] Jack C.R., Knopman D.S., Jagust W.J., Shaw L.M., Aisen P.S., Weiner M.W., Petersen R.C., Trojanowski J.Q. (2010). Hypothetical model of dynamic biomarkers of the Alzheimer’s pathological cascade. Lancet Neurol..

[8] Araujo J., Cai J., Stevens J. (2019). Prevalence of optimal metabolic health in American adults: National Health and Nutrition Examination Survey 2009–2016. Metab. Syndr. Relat. Disord..

[9] Luchsinger J.A., Tang M.-X., Shea S., Mayeux R. (2004). Hyperinsulinemia and risk of Alzheimer disease. Neurology.

[10] Young S.E., Mainous A.G., Carnemolla M. (2006). Hyperinsulinemia and cognitive decline in a middle-aged cohort. Diabetes Care.

[11] Craft S. (2009). The role of metabolic disorders in Alzheimer disease and vascular dementia: Two roads converged. Arch. Neurol..

[12] Razay G., Vreugdenhil A., Wilcock G. (2007). The metabolic syndrome and Alzheimer disease. Arch. Neurol..

[13] Whitmer R.A., Gunderson E.P., Barrett-Connor E., Quesenberry C.P., Yaffe K. (2005). Obesity in middle age and future risk of dementia: A 27 year longitudinal population based study. BMJ.

[14] Wei Z., Koya J., Reznik S.E. (2021). Insulin resistance exacerbates Alzheimer disease via multiple mechanisms. Front. Neurosci..

[15] Zhang W., Xiao D., Mao Q., Xia H. (2023). Role of neuroinflammation in neurodegeneration development. Signal Transduct. Target. Ther..

[16] Henriques J.F., Serra D., Dinis T.C.P., Almeida L.M. (2020). The Anti-Neuroinflammatory Role of Anthocyanins and Their Metabolites for the Prevention and Treatment of Brain Disorders. Int. J. Mol. Sci..

[17] Lail H.L., Feresin R.G., Hicks D., Stone B., Price E., Wanders D. (2021). Berries as a treatment for obesity-induced inflammation: Evidence from preclinical models. Nutrients.

[18] Basu A., Lyons T.J. (2011). Strawberries, blueberries, and cranberries in the metabolic syndrome: Clinical perspectives. J. Agric. Food Chem..

[19] Burton-Freeman B. (2010). Postprandial metabolic events and fruit-derived phenolics: A review of the science. Br. J. Nutr..

[20] Vendrame S., Del Bo’ C., Ciappellano S., Riso P., Klimis-Zacas D. (2016). Berry fruit consumption and metabolic syndrome. Antioxidants.

[21] Miller M.G., Shukitt-Hale B. (2012). Berry fruit enhances beneficial signaling in the brain. J. Agric. Food Chem..

[22] Winter A.N., Bickford P.C. (2019). Anthocyanins and their metabolites as therapeutic agents for neurodegenerative disease. Antioxidants.

[23] Marques C., Fernandes I., Meireles M., Faria A., Spencer J.P.E., Mateus N., Calhau C. (2018). Gut microbiota modulation accounts for the neuroprotective properties of anthocyanins. Sci. Rep..

[24] Basu A., Izuora K., Betts N.M., Kinney J.W., Salazar A.M., Ebersole J.L., Scofield R.H. (2021). Dietary strawberries improve cardiometabolic risks in adults with obesity and elevated serum LDL cholesterol in a randomized controlled crossover trial. Nutrients.

[25] Basu A., Izuora K., Hooyman A., Scofield H.R., Ebersole J.L. (2023). Dietary strawberries improve serum metabolites of cardiometabolic risks in adults with features of the metabolic syndrome in a randomized controlled crossover trial. Int. J. Mol. Sci..

[26] Cuomo P., Capparelli R., Iannelli A., Iannelli D. (2022). Role of branched-chain amino acid metabolism in type 2 diabetes, obesity, cardiovascular disease and non-alcoholic fatty liver disease. Int. J. Mol. Sci..

[27] Edirisinghe I., Banaszewski K., Cappozzo J., Sandhya K., Ellis C.L., Tadapaneni R., Kappagoda C.T., Burton-Freeman B.M. (2011). Strawberry anthocyanin and its association with postprandial inflammation and insulin. Br. J. Nutr..

[28] Burton-Freeman B., Linares A., Hyson D., Kappagoda T. (2010). Strawberry modulates LDL oxidation and postprandial lipemia in response to high-fat meal in overweight hyperlipidemic men and women. J. Am. Coll. Nutr..

[29] Huang L., Xiao D., Zhang X., Sandhu A.K., Chandra P., Kay C., Edirisinghe I., Burton-Freeman B. (2021). Strawberry consumption, cardiometabolic risk factors, and vascular function: A randomized controlled trial in adults with moderate hypercholesterolemia. J. Nutr..

[30] Joseph J., Shukitt-Hale B., Denisova N.A., Bielinski D., Martin A., McEwen J.J., Bickford P.C. (1999). Reversals of age-related declines in neuronal signal transduction, cognitive, and motor behavioral deficits with blueberry, spinach, or strawberry dietary supplementation. J. Neurosci..

[31] Shukitt-Hale B., Bielinski D.F., Lau F.C., Willis L.M., Carey A.N., Joseph J.A. (2015). The beneficial effects of berries on cognition, motor behaviour and neu-ronal function in ageing. Br. J. Nutr..

[32] Williams C.M., El Mohsen M.A., Vauzour D., Rendeiro C., Butler L.T., Ellis J.A., Whiteman M., Spencer J.P. (2008). Blueberry-induced changes in spatial working memory correlate with changes in hippocampal CREB phosphorylation and brain-derived neurotrophic factor (BDNF) levels. Free. Radic. Biol. Med..

[33] Devore E., Kang H., Breteler M., Grodstein F. (2012). Dietary intakes of berries and flavonoids in relation to cognitive decline. Ann. Neurol..

[34] Agarwal P., Holland T.M., Wang Y., Bennett D.A., Morris M.C. (2019). Association of strawberries and anthocyanidin intake with Alzheimer’s dementia risk. Nutrients.

[35] Miller M.G., Thangthaeng N., Rutledge G.A., Scott T.M., Shukitt-Hale B. (2021). Dietary strawberry improves cognition in a randomised, double-blind, placebo-controlled trial in older adults. Br. J. Nutr..

[36] Tsang M., Delaney K., Kern M., Jason N., Hong M.Y., Liu C., Hooshmand S. (2023). The impact of strawberries on cognition and cardiovascular health of older healthy adults: A randomized, crossover, double-blind, placebo controlled clinical trial. Curr. Dev. Nutr..

[37] Krikorian R., Zimmerman M.E., Fleck D.E. (2004). Inhibitory control in obsessive-compulsive disorder. Brain Cogn..

[38] Stein A.L., Tolle K.A., Stover A.N., Shidler M.D., Krikorian R. (2023). Detecting mild cognitive impairment remotely with the modified memory impairment screen by telephone. Neuropsychol. Dev. Cogn. B Aging Neuropsychol. Cogn..

[39] Taves D.R. (1974). Minimization: A new method of assigning patients to treatment and control groups. Clin. Pharmacol. Ther..

[40] Porteus S.D. (1965). Porteus Maze Test: Fifty Years’ Application.

[41] Krikorian R., Bartok J.A. (1998). Developmental Data for the Porteus Maze Test. Clin. Neuropsychol..

[42] Reitan R.M. (1992). Trail Making Test: Manual for Administration and Scoring.

[43] Sánchez-Cubillo I., Periáñez J., Adrover-Roig D., Rodríguez-Sánchez J., Ríos-Lago M., Tirapu J., Barceló F. (2009). Construct validity of the Trail Making Test: Role of task-switching, working memory, inhibition/interference control, and visuomotor abilities. J. Int. Neuropsychol. Soc..

[44] Welsh M.C., Pennington B.F., Groisser D.B. (1991). A normative-developmental study of executive function: A window on prefrontal function in children. Dev. Neuropsychol..

[45] Shao Z., Janse E., Visser K., Meyer A.S. (2014). What do verbal fluency tasks measure? Predictors of verbal fluency performance in older adults. Front. Psychol..

[46] Miceli G., Caltagirone C., Gainotti G., Masullo C., Silveri M.C. (1981). Neuropsychological correlates of localized cerebral lesions in non-aphasic brain-damaged patients. J. Clin. Neuropsychol..

[47] Delis D.C., Kramer J.H., Kaplan E., Ober B.A. (2000). California Verbal Learning Test—Second Edition (CVLT—II).

[48] Krikorian R. (1996). Independence of verbal and spatial paired associate learning. Brain Cogn..

[49] Beck A.T., Steer R.A., Brown G.K. (1996). BDI-II: Beck Depression Inventory Manual.

[50] Wang Y.-P., Gorenstein C. (2013). Psychometric properties of the Beck Depression Inventory-II: A comprehensive review. Rev. Bras. Psiquiatr..

[51] Steer R.A., Ball R., Ranieri W.F., Beck A.T. (1999). Dimensions of the Beck Depression Inventory-II in clinically depressed outpa-tients. J. Clin. Psychol..

[52] Wallace T.M., Levy J.C., Matthews D.R. (2004). Use and abuse of HOMA modeling. Diabetes Care.

[53] Gong R., Luo G., Wang M., Ma L., Sun S., Wei X. (2021). Associations between TG/HDL ratio and insulin resistance in the US population: A cross-sectional study. Endocr. Connect..

[54] Moriyama K. (2020). Associations between the triglyceride to high-density lipoprotein cholesterol ratio and metabolic syndrome, insulin resistance, and lifestyle habits in healthy japanese. Metab. Syndr. Relat. Disord..

[55] da Luz P.L., Favarato D., Junior J.R.F.-N., Lemos P., Chagas A.C.P. (2008). High ratio of triglycerides to HDL-cholesterol predicts extensive coronary disease. Clinics.

[56] Krikorian R., Skelton M.R., Summer S.S., Shidler M.D., Sullivan P.G. (2022). Blueberry supplementation in midlife for dementia risk reduction. Nutrients.

[57] Cohen J. (1988). Statistical Power Analysis for the Behavioral Sciences.

[58] Riordan H.J. (2017). Constructing composites to optimize cognitive outcomes. J. Clin. Stud..

[59] Sheeber L.B., Sorensen E.D., Howe S.R. (1996). Data analytic techniques for treatment outcome studies with pretest/posttest measurements: An extensive primer. J. Psychiatr. Res..

[60] Verkouter I., Noordam R., le Cessie S., van Dam R.M., Lamb H.J., Rosendaal F.R., van Heemst D., de Mutsert R. (2019). The Association between Adult Weight Gain and Insulin Resistance at Middle Age: Mediation by Visceral Fat and Liver Fat. J. Clin. Med..

[61] Gutch M., Kumar S., Razi S.M., Gupta K.K., Gupta A. (2015). Assessment of insulin sensitivity/resistance. Indian J. Endocrinol. Metab..

[62] Qaseem A., Wilt T.J., Kansagara D., Horwitch C., Barry M.J., Forciea M.A., Clinical Guidelines Committee of the American College of Physicians (2018). Hemoglobin A_1c_ targets for glycemic control with pharmacologic therapy for nonpregnant adults with type 2 diabetes mellitus: A guidance statement update from the American college of physicians. Ann. Intern. Med..

[63] Babic N., Valjevac A., Zaciragic A., Avdagic N., Zukic S., Hasic S. (2019). The triglyceride/HDL ratio and triglyceride glucose index as predictors of glycemic control in patients with diabetes mellitus type. Med. Arch..

[64] Olivas-Aguirre F.J., Rodrigo-García J., Martínez-Ruiz N.D.R., Cárdenas-Robles A.I., Mendoza-Díaz S.O., Álvarez-Parrilla E., González-Aguilar G.A., De la Rosa L.A., Ramos-Jiménez A., Wall-Medrano A. (2016). Cyanidin-3-O-glucoside: Physical-Chemistry, Foodomics and Health Effects. Molecules.

[65] Atkins A.S., Berman M.G., Reuter-Lorenz P.A., Lewis R.L., Jonides J. (2011). Resolving semantic and proactive interference in memory over the short-term. Mem. Cogn..

[66] Wahlheim C.N. (2014). Proactive effects of memory in young and older adults: The role of change recollection. Mem. Cogn..

[67] Loewenstein D.A., Curiel R.E., Wright C., Sun X., Alperin N., Crocco E., Czaja S.J., Raffo A., Penate A., Melo J. (2017). Recovery from Proactive Semantic Interference in Mild Cognitive Impairment and Normal Aging: Relationship to Atrophy in Brain Regions Vulnerable to Alzheimer’s Disease. J. Alzheimer’s Dis..

[68] Kato T. (2021). Coping with stress, executive functions, and depressive symptoms: Focusing on flexible responses to stress. J. Clin. Med..

[69] Morris M.C., Evans L.D., Rao U., Garber J. (2014). Executive function moderates the relation between coping and depressive symptoms. Anxiety Stress Coping.

[70] Ferguson H.J., Brunsdon V.E.A., Bradford E.E.F. (2021). The developmental trajectories of executive function from adolescence to old age. Sci. Rep..

[71] Yang Y., Shields G.S., Guo C., Liu Y. (2018). Executive function performance in obesity and overweight individuals: A me-ta-analysis and review. Neurosci. Biobehav. Rev..

[72] Lutski M., Weinstein G., Goldbourt U., Tanne D. (2017). Insulin resistance and future cognitive performance and cognitive decline in elderly patients with cardiovascular disease. J. Alzheimer’s Dis..

[73] Morys F., Potvin O., Zeighami Y., Vogel J., Lamontagne-Caron R., Duchesne S., Dagher A., Initiative F.T.A.D.N. (2023). Obesity-Associated Neurodegeneration Pattern Mimics Alzheimer’s Disease in an Observational Cohort Study. J. Alzheimer’s Dis..

[74] Willmann C., Brockmann K., Wagner R., Kullmann S., Preissl H., Schnauder G., Maetzler W., Gasser T., Berg D., Eschweiler G.W. (2020). Insulin sensitivity predicts cognitive decline in individuals with prediabetes. BMJ Open Diabetes Res. Care.

[75] Raji C.A., Meysami S., Hashemi S., Garg S., Akbari N., Gouda A., Chodakiewitz Y.G., Nguyen T.D., Niotis K., Merrill D.A. (2023). Visceral and subcutaneous abdominal fat predict brain volume loss at midlife in 10,001 individuals. Aging Dis..

[76] Schmoller A., Hass T., Strugovshchikova O., Melchert U.H., Scholand-Engler H.G., Peters A., Schweiger U., Hohagen F., Oltmanns K.M. (2010). Evidence for a relationship between body mass and energy metabolism in the human brain. J. Cereb. Blood Flow Metab..

[77] Salehi B., Sharifi-Rad J., Cappellini F., Reiner Ž., Zorzan D., Imran M., Sener B., Kilic M., El-Shazly M., Fahmy N.M. (2020). The therapeutic potential of anthocyanins: Current approaches based on their molecular mechanism of action. Front. Pharmacol..

[78] Skrovankova S., Sumczynski D., Micke J., Jurikova T., Sochor J. (2015). Bioactive compounds and antioxidant activity in different types of berries. Int. J. Mol. Sci..

[79] Andres-Lacueva C., Shukitt-Hale B., Galli R.L., Jauregui O., Lamuela-Raventos R.M., Joseph J.A. (2005). Anthocyanins in aged blueberry-fed rats are found centrally and may enhance memory. Nutr. Neurosci..

[80] Rendeiro C., Vauzour D., Rattray M., Waffo-Téguo P., Mérillon J.M., Butler L.T., Williams C.M., Spencer J.P.E. (2013). Dietary levels of pure flavonoids improve spatial memory performance and increase hippocampal brain-derived neurotrophic factor. PLoS ONE.

[81] Poulose S.M., Rabin B.M., Bielinski D.F., Kelly M.E., Miller M.G., Thanthaeng N., Shukitt-Hale B. (2017). Neurochemical differences in learning and memory paradigms among rats supplemented with anthocyanin-rich blueberry diets and exposed to acute doses of 56Fe particles. Life Sci. Space Res..

[82] Shukitt-Hale B., Carey A.N., Jenkins D., Rabin B.M., Joseph J.A. (2007). Beneficial effects of fruit extracts on neuronal function and behavior in a rodent model of accelerated aging. Neurobiol. Aging.

[83] Carey A.N., Gomes S.M., Shukitt-Hale B. (2014). Blueberry supplementation improves memory in middle-aged mice fed a high-fat diet. J. Agric. Food Chem..

[84] Krikorian R., Kalt W., McDonald J.E., Shidler M.D., Summer S.S., Stein A.L. (2019). Cognitive performance in relation to urinary anthocyanins and their flavonoid-based products following blueberry supplementation in older adults at risk for dementia. J. Funct. Foods.

